# A Rare Case of Tuberculosis as a Cause of Lytic Lesion of Talus Without Adjacent Bone Involvement in a Four-Year-Old Child

**DOI:** 10.7759/cureus.16909

**Published:** 2021-08-05

**Authors:** Samrat S Sahoo, Vivek Tiwari, Sandeep Velagada

**Affiliations:** 1 Orthopaedics, All India Institute of Medical Science Nagpur, Nagpur, IND; 2 Orthopaedics, Saheed Laxman Nayak Medical College and Hospital, Koraput, IND

**Keywords:** tuberculosis, talus, antitubercular therapy, debridement, foot tuberculosis, lytic lesion

## Abstract

Extra-pulmonary tuberculosis still remains an important differential diagnosis for chronic musculoskeletal ailments in developing countries like India and may involve any part of the body without characteristic systemic features. We are presenting a rare case of a four-year-old female child, who came to our tertiary-care hospital with chief complaints of pain in the left foot along with a gradually increasing swelling over the dorsum of the foot for the past five months. There was no history of trauma or constitutional symptoms. The serum inflammatory markers were found raised, and X-ray and magnetic resonance imaging revealed an isolated lytic lesion in the talus bone. Debridement, as well as curettage of the lesion, was done, both as a diagnostic and therapeutic procedure. A caseous cheesy material was evacuated and sent for microbiological and histopathological evaluation, which revealed the presence of acid-fast bacilli and granulomatous lesion confirming the diagnosis of tuberculosis. The patient was started with anti-tubercular chemotherapy, which continued for a total duration of 14 months, along with foot and ankle immobilization in a below-knee cast for three months. After completion of therapy, there was complete resolution of the lytic lesion on x-ray, with full symptom relief, and a full range of movement of the ankle was obtained. In cases with longstanding pain and swelling of the foot, with or without associated systemic symptoms, tuberculosis should be considered as a strong differential diagnosis even in young children, especially in developing countries. Diagnostic and therapeutic curettage along with anti-tubercular chemotherapy can result in a good functional outcome in such patients.

## Introduction

In developing countries including India, the high prevalence of tuberculosis including extra-pulmonary tuberculosis is a major health concern. The patients with musculoskeletal involvement constitute around 1-3% of the total case-load of tuberculosis [1]. Although the spine is the most common site of musculoskeletal involvement, tuberculosis may involve any bone and joint with varied presentations. After the spine, the most commonly involved regions include large weight-bearing joints like the hip and knee. The incidence of tuberculosis infection in the foot and ankle bones and joints is very small [1-3]. We present a rare case of musculoskeletal tuberculosis involving talus bone without any adjacent bone or joint involvement in a four-year-old child.

## Case presentation

**Figure 1:**
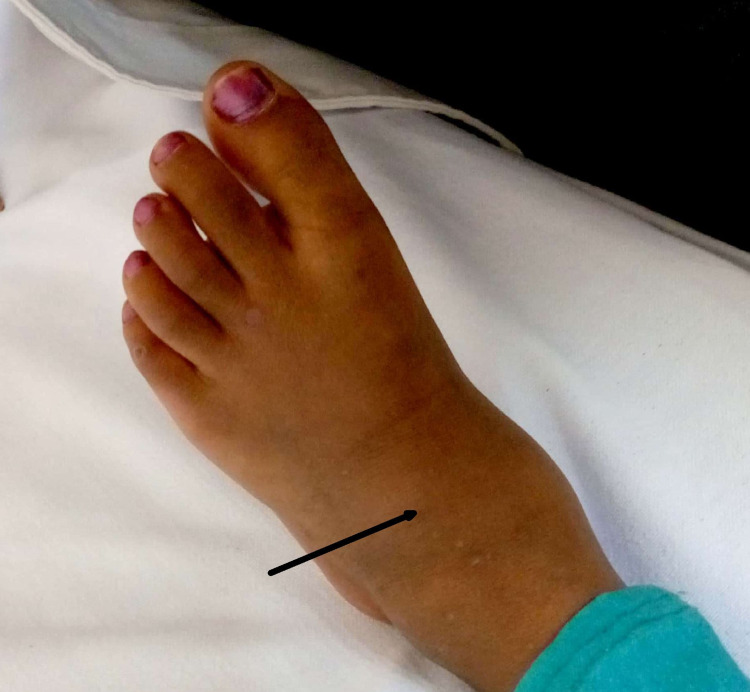
Clinical picture of the left foot at presentation There was mild swelling seen over the dorsum of the left foot (black arrow).

A four-year-old female child came to our hospital with chief complaints of pain and swelling over the left foot for the past five months. The intensity of pain increased on prolonged walking, running, or playing, and the foot swelling was gradually increasing in size. There was no history of fever, weight loss, or other constitutional symptoms. Also, there was no antecedent history of trauma. On clinical examination, there was localized mild tenderness and swelling over the dorsum of the left foot (Figure 1); however, the ankle range of motion was full with pain on extreme dorsiflexion.

**Figure 2:**
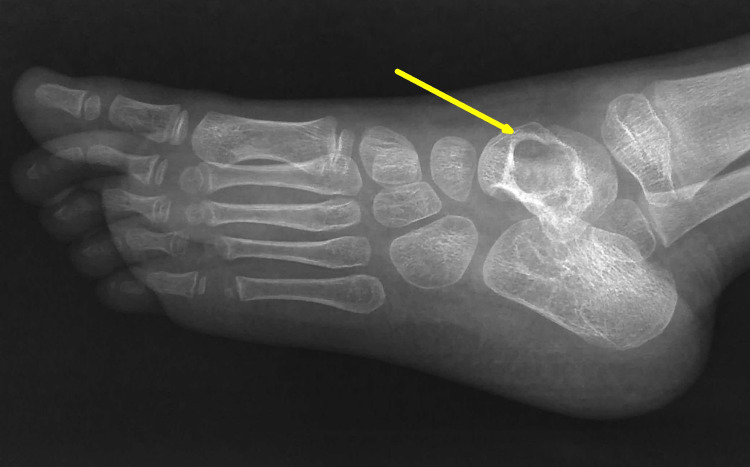
Plain x-ray of left ankle lateral view at presentation The x-ray showed an isolated lytic lesion in the talus bone with a sclerotic rim without any cortical breach (yellow arrow).

Routine hematological investigations including complete blood count, erythrocyte sedimentation rate (ESR), C-reactive protein (CRP), and Mantoux test were advised. The serum inflammatory markers were raised with ESR 75 mm/hour and CRP 50 mg/dl, and the Mantoux test was found positive. A plain radiograph of the left ankle joint revealed an isolated lytic lesion in the talus bone with a sclerotic rim (Figure 2).

**Figure 3:**
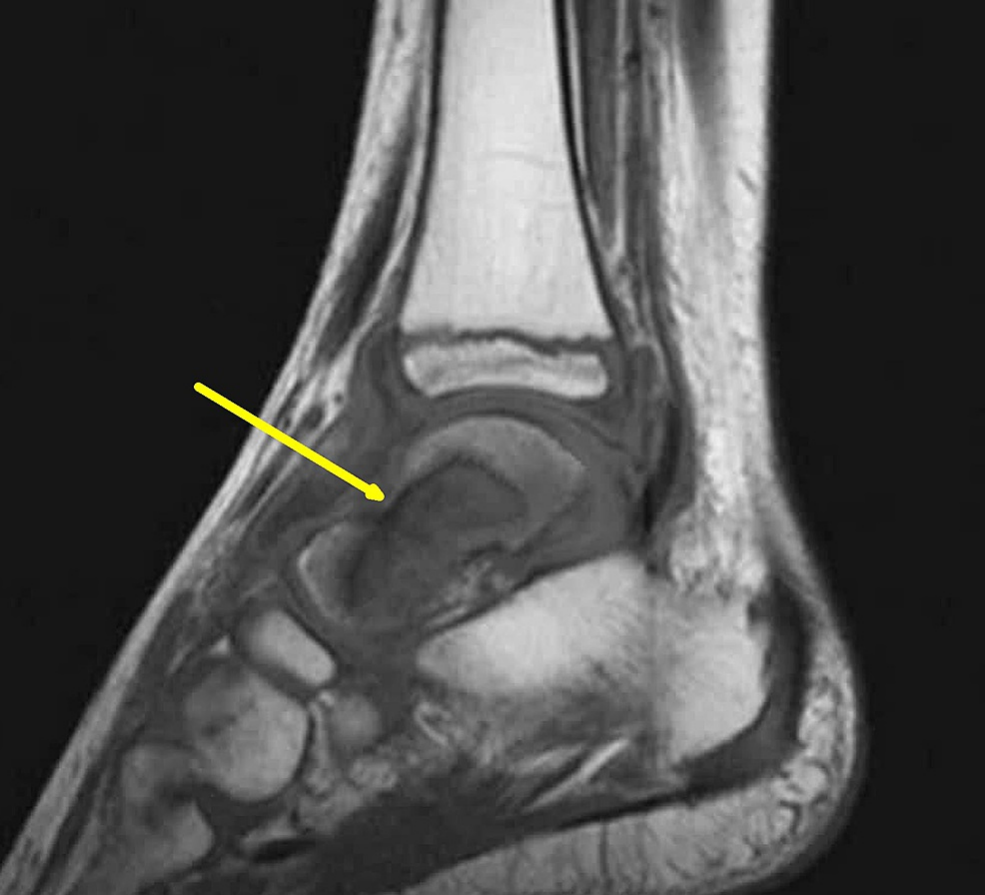
Magnetic resonance imaging of left ankle at presentation The T1 weighted image shows a hypointense lesion in the talus bone with surrounding marrow edema without any cortical breach (yellow arrow).

Magnetic resonance imaging of the left ankle showed a hypointense lesion in the talus on T1 weighted images with surrounding marrow edema, and also mild synovial thickening was seen in the tibiotalar joint. There was no soft tissue mass, fluid/hemorrhagic level, or any cortical breach in the talus (Figure 3).

**Figure 4:**
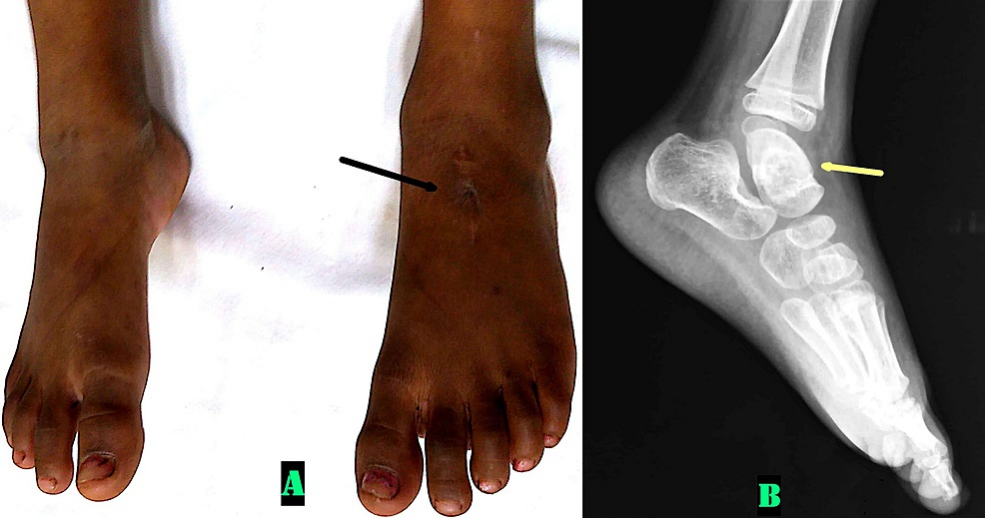
Clinical and x-ray picture at final follow-up. 4A: Clinical picture of both feet showing healed surgical scar on the dorsum of left foot (black arrow), 4B: Plain radiograph of left ankle lateral view showing complete resolution of the lesion (yellow arrow)

Debridement and curettage of the lesion were done both as a diagnostic and therapeutic procedure. During surgery, caseous cheesy material came out from the talus, which was sent for Gram staining, Zeihl-Neelsen staining, histopathology, and culture. The Zeihl-Neelsen staining showed acid-fast bacilli, and the histopathology revealed caseous granulation tissue. The patient’s foot and ankle were immobilized in posterior slab support for two weeks till suture removal, which was then replaced with a plaster cast. The cast was removed after three months, after which active and passive ankle range of motion exercises were started. After histological confirmation, the anti-tubercular chemotherapy was started. It consisted of initial two months of four-drug therapy (intensive phase)--isoniazid (H) (10 mg/kg), rifampicin (R) (15mg/kg), pyrazinamide (Z) (35 mg/kg), and ethambutol (E) (20 mg/kg)--followed by 12 months of two-drug therapy (HR) (continuation phase). The patient was followed every two weeks after the start of treatment, up to the end of the intensive phase, and then every two months until completion of treatment. The parameters assessed on follow-up visits included symptomatic improvement, adherence to treatment, inquiry about any adverse events, and weight measurement. Dosages of drugs were adjusted to take account of any weight gain [4]. She was also periodically evaluated for radiological improvement of the lesion. After completion of therapy, there was complete resolution of the lytic lesion, with the patient playing happily without any pain with a full range of ankle movement (Figure 4).

## Discussion

Tuberculosis involving either the pulmonary or extrapulmonary region is still a major healthcare burden in developing countries like India. Among the extra-pulmonary cases of tuberculosis, musculoskeletal involvement accounts for 1-3% of the caseload, out of which 30-50% involve axial skeleton (spinal tuberculosis) [1]. Tubercular involvement of hip and knee is also relatively common, however, foot and ankle disease accounts for only 1% of all musculoskeletal tuberculosis infections [1,3]. Due to their rare presentation along with the absence of typical constitutional symptoms of tuberculosis, there is a delay in diagnosis and initiation of the treatment [5,6]. Early prompt diagnosis depends on clinical suspicion and thorough clinic-radiological evaluation.

The description of isolated tuberculosis of talus bone is relatively rare in English literature. Moreover, such presentation in a child below five years of age is sparsely reported. A case of tubercular involvement of talus bone was reported by Haraldsson in a two-and-a-half-year-old child after receiving Bacillus Calmette-Guérin vaccine [7]. Anderson et al. described a case of talus tuberculosis in a six-year-old child, with simultaneous involvement of the fifth lumbar vertebra and left kidney, with substantial delay in the diagnosis [3]. The isolated tubercular disease of the talus was also reported in an Asian-origin 29-year-old patient in Germany, which required partial talectomy with ankle fusion [8]. Boussouga et al. reported a case of talus tuberculosis in an elderly woman who was diagnosed using biopsy and successfully treated conservatively using three-drug anti-tubercular chemotherapy for 12 months, though the patient also developed reflex sympathetic dystrophy [2].

Anand et al. also described a similar case of tuberculosis involving talus bone which presented as a lytic lesion; the lesion was treated with curettage and bone grafting [9]. Our case was successfully treated using debridement and curettage alone, along with the complete course of anti-tubercular chemotherapy for 14 months. Similar to our case, Dahuja et al. reported tubercular involvement of talus in a 14-year-old boy who was treated with debridement and curettage, with anti-tubercular therapy [10]. The trans-malleolar approach has also been reported for curettage and debridement of such cases [11]. We did not require an osteotomy and approached the talus through the anterior aspect of the foot. Karkhur et al. also described a case of talar tuberculosis in a 20-year-old man which was treated conservatively with anti-tubercular therapy with good outcomes, after histopathological confirmation of the diagnosis [12].

Our patient presented with complaints of foot pain and swelling for the past five months; the diagnosis was significantly delayed due to the lack of awareness as well as due to the lack of classical constitutional symptoms of tuberculosis. The presence of a long-standing lytic lesion pointed towards the diagnosis of either a benign tumor or a chronic-infective etiology; the diagnosis was confirmed after the staining and histopathology of the curetted material. Debridement and curettage helped both as a diagnostic and therapeutic intervention, confirming the diagnosis and evacuation of the caseous material [5]. Elevated ESR and CRP levels add to the clinical suspicion of tuberculosis, and also act as a marker for serially assessing the resolution of disease activity. The child achieved an excellent outcome after completion of the anti-tubercular therapy, and the x-ray confirmed the full resolution of the lytic lesion.

## Conclusions

Tuberculosis should be considered as a differential diagnosis in pediatric patients presenting with chronic pain and swelling of the foot. Identification of a lytic lesion on x-ray needs to be thoroughly evaluated using serum inflammatory markers as well as advanced imaging investigations including magnetic resonance imaging. Debridement and curettage help in obtaining tissue diagnosis as well as therapeutic clearance of the infected and necrotic tissues in these cases. Such patients can achieve good outcomes after completion of the full course of anti-tubercular chemotherapy, along with local protection and support using cast immobilization in the initial phase of treatment.
